# Mediastinal chyloma after lung cancer surgery: case report

**DOI:** 10.1186/s13019-016-0522-z

**Published:** 2016-08-02

**Authors:** Masashi Furukawa, Hiroyuki Tao, Toshiki Tanaka, Kazunori Okabe

**Affiliations:** Division of Thoracic Surgery, National Hospital Organization Yamaguchi-Ube Medical Center, 685 Higashikiwa, Ube, Yamaguchi 755-0241 Japan

**Keywords:** Chylothorax, Chyloma, Lung cancer, Pleurodesis

## Abstract

**Background:**

Chylothorax is a relatively rare but well-known complication of thoracic surgery.

**Case Presentation:**

A 70-year-old man underwent right upper and middle bilobectomy and systematic lymph node dissection through a posterolateral thoracotomy for lung cancer. On the second postoperative day, he developed chylothorax that was treated with dietary management and pleurodesis. The discharge diminished and his chest tube was removed on the ninth postoperative day. On the 14^th^ postoperative day, the patient complained of dyspnea and dysphagia, and imaging studies revealed mediastinal chyloma. Thoracoscopic surgical drainage was performed and the site of chyle leakage was sutured.

**Conclusions:**

This report presents an unexpected complication of chemical pleurodesis and reviews the indications for surgical intervention in cases of postoperative chylothorax.

## Background

Chylothorax is a relatively rare but well-known complication of thoracic surgery. It is treated with some conservative approaches such as oral intake cessation including nil per os with total parenteral nutrition, low-fat diet management, and chemical pleurodesis [1]. Surgical intervention is considered only when conservative treatments have failed. We report a case of mediastinal chyloma possibly induced by pleurodesis to treat chylothorax following lung cancer surgery.

## Case presentation

**Fig. 1:**
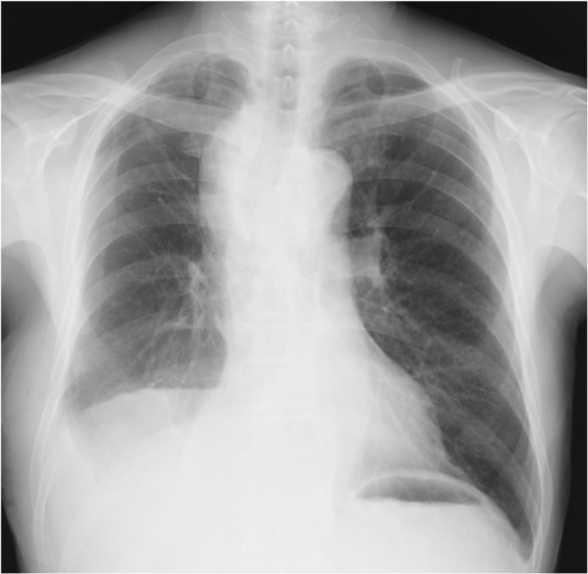
Chest radiography showing upper mediastinal mass

**Fig. 2:**
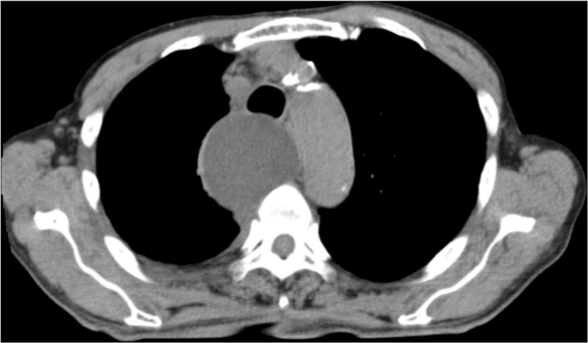
Chest computed tomography showing a large mediastinal fluid collection surrounded by a thick capsule that is compressing the trachea and esophagus

**Fig. 3:**
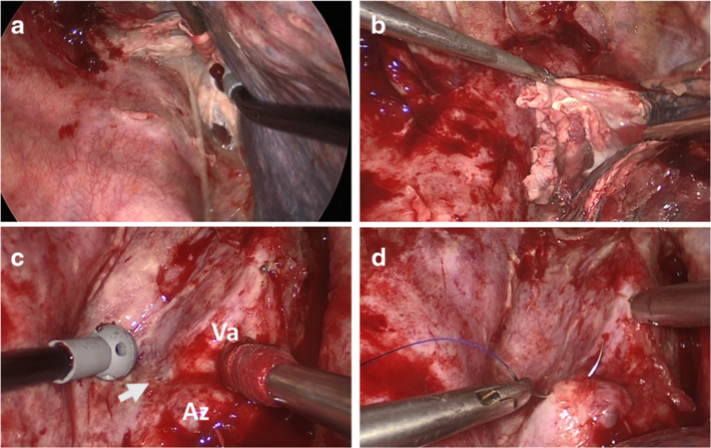
Intra-operative image of the second surgery. When the thick capsule was incised, chyle began to spill out (**a**). thick capsule (**b**). The point of chylous leakage near the azygos vein and vagus nerve (**c**). Repairing the leakage point using a Z-suture with 4–0 Prolene (**d**). Arrow mark indicating the point of chylous leakage. Az indicating azygos vein, Va indicating vagus nerve

A 70-year-old man was diagnosed with lung adenocarcinoma (clinical T4N0M0) involving a right upper lobe mass of 4.0 cm in diameter and a metastatic nodule in the middle lobe. He underwent right upper and middle bilobectomy and systematic lymph node dissection through a posterolateral thoracotomy. On the second postoperative day, 1750 mL of white turbid fluid was drained through the chest tube and the effusion was diagnosed as chylothorax. During the first 24 h following implementation of a fat-free dietary regimen, the color of the discharge became transparent, but another 630 mL of discharge was observed. Although the amount of discharge reduced gradually, it was persistent. Chemical pleurodesis was performed on the sixth postoperative day for which 5 KE of OK-432 (Picibanil; Chugai Pharmaceutical, Tokyo, Japan) and 300 mg of minocycline were administered into the thoracic cavity. The discharge began to progressively diminish. Three days thereafter, the amount of discharge had decreased to 150 mL/day, and on the ninth postoperative day, the chest tube was removed. On the 14^th^ postoperative day, the patient complained of dyspnea and dysphagia, and an upper mediastinal mass was detected on chest radiogram (Fig. 1). Chest computed tomography revealed a large mediastinal fluid collection 6 cm in diameter surrounded by a thick capsule that was compressing the trachea and esophagus (Fig. 2). The mass was suspected to be a mediastinal chyloma. Since the patient’s symptoms were gradually progressing, we performed surgery on the 15^th^ postoperative day. Thirty minutes before the surgery, ice cream was given to the patient to help confirm the leakage point in the thoracic duct. Thoracoscopy revealed a small amount of chylous effusion in the thoracic cavity and an elastic mass in the upper mediastinum. When the thick capsule was incised, chyle began to spill out (Fig. 3a, b). Once the chyle had drained completely, we confirmed the point of leakage and repaired it using a Z-suture with 4–0 Prolene (Ethicon, Somerville, NJ) (Fig. 3c, d). The postoperative course was uneventful and the patient was free of any symptoms. He remained asymptomatic 12 months after discharge from the hospital.

## Discussion

Chylothorax after lung cancer surgery is relatively rare, occurring in only 2.2–2.4 % of cases [1–3]. Management strategies for chylothorax have been well discussed, but so far there is no consensus as to protocol. Traditionally, chylothorax is treated conservatively with dietary modification. The basic principle of conservative treatment for chylothorax is to inflate the remnant lung to decrease the dead space and to promote spontaneous adhesion around the injured thoracic duct [2]. Cerfolio et al. [4] recommended observation of the patient for seven days with dietary management. At that point, if the drainage is still greater than 1000 mL/day, reoperation to ligate the thoracic duct is necessary. In cases where dietary management are insufficient, pleurodesis must be considered. Since the amount of chylous fluid may reduce the efficacy of pleurodesis, Shimizu et al. [2] recommended early surgical intervention if chest tube drainage of more than 500 mL of chylous fluid was observed during the first 24 h after complete oral intake cessation and the initiation of total parenteral nutrition. It should also be noted that pleurodesis has the potential to make subsequent surgery more difficult. In fact, in this case we found several fibrous adhesions around the remnant lower lobe during the second surgery, which could eventually lead to chyloma formation. Suzuki et al. [5] likewise reported a case of a mediastinal chyloma after a conservative treatment with OK-432 following right upper lobectomy and systematic lymph node dissection. They reported that the thick capsule of the chyloma may have been the results of severe inflammation response caused by the intrapleural injection of OK-432. Their report and our case both suggest that pleurodesis may fail to promote proper adhesion around the injured thoracic duct when it is difficult to inflate the remnant lung because a large amount of chylous discharge is located in the dead space. If there are some chyle flow rate and pressure, once the growth of fibrous adhesions following pleurodesis blocks chylous discharge from entering the chest tube, occult chyloma could arise despite a seemingly successful pleurodesis with apparent cessation of drainage.

## Conclusion

This report presents the serious complications and limitations of chemical pleurodesis and reviews the indications for surgical intervention in postoperative chylothorax. Although mediastinal chyloma is quite rare, it is important to note that it can develop following chemical pleurodesis treatment for chylothorax with a large amount of discharge and that it can present symptoms due to compression of the trachea and/or the esophagus.
